# COVID-19-related future anxiety is associated with the health-related quality of life in school-aged children and adolescents—A cross-sectional study

**DOI:** 10.3389/fpubh.2022.1003876

**Published:** 2022-11-09

**Authors:** Anika Kästner, Petra Lücker, Arne Hannich, Lena Schmeyers, Janny Lücker, Wolfgang Hoffmann

**Affiliations:** Institute for Community Medicine, Section Epidemiology of Health Care and Community Health, University Medicine Greifswald, Greifswald, Germany

**Keywords:** school children, health-related quality of life (HRQL), mental health, COVID-19-related future anxiety, KIDSCREEN-10, Dark Future Scale for children

## Abstract

**Background:**

Over the course of the COVID-19 pandemic, previous studies have shown that the physical as well as the mental health of children and adolescents significantly deteriorated. Future anxiety caused by the COVID-19 pandemic and its associations with quality of life has not previously been examined in school children.

**Methods:**

As part of a cross-sectional web-based survey at schools in Mecklenburg-Western Pomerania, Germany, two years after the outbreak of the pandemic, school children were asked about COVID-19-related future anxiety using the German epidemic-related Dark Future Scale for children (eDFS-K). Health-related quality of life (HRQoL) was assessed using the self-reported KIDSCREEN-10. The eDFS-K was psychometrically analyzed (internal consistency and confirmatory factor analysis) and thereafter examined as a predictor of HRQoL in a general linear regression model.

**Results:**

A total of *N* = 840 8–18-year-old children and adolescents were included in the analysis. The eDFS-K demonstrated adequate internal consistency reliability (Cronbach's α = 0.77), and the confirmatory factor analysis further supported the one-factor structure of the four-item scale with an acceptable model fit. Over 43% of students were found to have low HRQoL. In addition, 47% of the students sometimes to often reported COVID-19-related fears about the future. Children with COVID-19-related future anxiety had significantly lower HRQoL (*B* = – 0.94, *p* < 0.001). Other predictors of lower HRQoL were older age (*B* = – 0.63, *p* < 0.001), and female (*B* = – 3.12, *p* < 0.001) and diverse (*B* = – 6.82, *p* < 0.001) gender.

**Conclusion:**

Two years after the outbreak of the pandemic, school-aged children continue to exhibit low HRQoL, which is further exacerbated in the presence of COVID-19-related future anxiety. Intervention programs with an increased focus on mental health also addressing future anxiety should be provided.

## Introduction

Long after the COVID-19 outbreak, the pandemic continues to impact our everyday lives with new emerging SARS-CoV-2 variants, frequently changing hygiene requirements, and contact restrictions. However, it is not only the impact of COVID-19 as a disease, but also the psychosocial consequences of lockdowns and contact restrictions in particular that had an impact on society. As previous studies have shown, children and adolescents worldwide suffered massive physical and psychological burdens as a result of the restrictions imposed by the pandemic (1–4). In addition to school closures and lack of exercise, it was above all the restrictions on social life that impaired children's and adolescents' everyday lives (5). Several risk factors for increased psychosocial distress were identified, such as low socioeconomic status, small living space, chronic physical conditions and mental stress on parents due to a job loss, or preexisting mental problems, whereas social and family support, along with a positive coping style, were associated with better mental health outcomes (2, 6–11).

One systematic review by Viner et al. investigating studies conducted during the first COVID-19 wave from February to July 2020 concluded that studies of short-term school closures reported adverse mental health symptoms and health behaviors among children and adolescents (3). In Germany, nationwide cross-sectional and longitudinal studies performed during the COVID-19 pandemic in children and adolescents found a significantly reduced health-related quality of life (HRQoL), more mental health problems, and higher anxiety levels during the pandemic (12–15).

The pandemic affected all areas of life and led in many ways to increased uncertainty about the future, e.g., regarding travel plans, leisure activities, participation in social and cultural events (such as theater, concerts, weddings), the pursuit of hobbies, but also regarding the employment perspective: Many sectors (e.g., tourism, event industry) have been restructured in the context of the pandemic, employment perspectives have worsened for a variety of professions, and many workers were laid off or put on short-time work. If the perspective on a positive future is overshadowed by worries, this can cause future anxiety. According to Zaleski, future anxiety is a state of apprehension, uncertainty, fear, worry, and concerns about unfavorable changes in the future, whereby the future refers to a greater temporal distance (16). In this context, future anxiety does not only refer to fears of specific future events, but also to the general perception of the personal future with crises, difficulties, the non-achievement of important goals and social aspects (16, 17). Here, cognitive and emotional processes, such as thoughts, associations, and fantasies regarding the future are stimuli for future anxiety of which people are fully aware (conscious anxiety) (16).

Previous studies have examined future anxiety and the impact of health literacy, particularly among college students (18, 19). It was found that higher health literacy was associated with lower future anxiety among Polish adults (18). Furthermore, one survey in Germany comprising over 14,000 university students showed that high levels of future anxiety were associated with low/very low wellbeing (20). Another study from Norway found no effect of COVID-19-related worries (worries about infecting others with COVID-19 and worries about family / friends becoming sick) in adolescents (16–18 years olds) on HRQoL (21). However, to our knowledge until to date, no study has examined future anxiety in children and adolescents and its influence on HRQoL.

The primary aim of this study was therefore to examine the association of COVID-19-related future anxiety and HRQoL in children and adolescents.

## Methods

### Study design and sample

As part of the project schugi-MV (scientific support of school opening in Mecklenburg-Western Pomerania), a cross-sectional online survey of students in Mecklenburg-Western Pomerania, Germany was conducted from 11th of February until 07th of March 2022. The primary aim of the study was to examine the HRQoL of school-aged children and adolescents in relation to future anxiety associated with the COVID-19 pandemic. The study was approved by the ethics committee of the University Medicine Greifswald (BB 163/21).

### Study region

The study was conducted in the northern German state Mecklenburg-Western Pomerania. At the time of the survey, two years after the first COVID-19 pandemic outbreak, the 7-day incidence in the region averaged at 3,262 PCR confirmed infections among 6–11-year olds and at 2,597 among 12–17-year olds [Supplementary Figure 1, (22)]. During the study period, school attendance was compulsory. On 07th of March 2022, the mask requirement during classes was lifted for all grade levels, but was still in effect in the school building outside of the classrooms until 25th of April. Mandatory rapid antigen tests in schools were conducted three times per week until 29th of April 2022.

### Implementation of the survey

The web-based survey was conducted using SoSci Survey (23). In addition to demographic variables such as age, gender, grade level, and type of school, the self-reported KIDSCREEN-10 was applied to assess the HRQoL and the epidemic-related Dark Future Scale for children (eDFS-K) was used to assess COVID-19-related future anxiety. With regard to the assessment of the type of school, there are different secondary school types in the study region with varying length of education: regional school (5th−10th grade), grammar school (5th−12th grade) and comprehensive school (5th−10th grade and 5th−12th grade). The general university entrance qualification can be achieved at grammar schools and comprehensive schools upon completion of the 12th grade. Gender could be specified as female, male or diverse. The KIDSCREEN-10 was applied under a cooperative agreement with the KIDSCREEN group (24–26). The eDFS-K is available for non-commercial research purposes by ZIS (license CC-BY-NC-SA-4.0), an open access repository for social and behavioral science measurement instruments operated by GESIS—Leibniz Institute for Social Sciences (27).

The links to the online survey were sent to all general schools in Mecklenburg-Western Pomerania (total of *N*=134,505 students at general schools in 2020/2021) by the responsible Ministry of Education, Science and Culture Mecklenburg-Western Pomerania and were forwarded to parents and students by the school principals and teachers. There was no direct contact between the study team and the participants. The introductory text of the survey indicated that parents should consent to the child's participation, however, the children were encouraged to complete the questionnaire themselves. A reminder was sent out once during the study period. The online survey was conducted anonymously. No personal identifying data was collected and no IP addresses were stored. It was not possible to trace the data back to individuals or schools.

### Self-reported KIDSCREEN-10

A standardized, validated, and internationally recognized instrument was used to measure the HRQoL (28). The self-reported KIDSCREEN-10 Index consists of 10 items and measures the general HRQoL in 8- to 18-year-olds (25). It includes questions such as “*Did you feel fit and well?*”, “*Did you feel sad?*”, or “*Did you do well at school?*”. All items are rated on a 5-point Likert scale with the options “never”, “rarely”, “sometimes”, “often”, and “always”. Based on these items, a T-score was calculated according to the developers' specifications with a mean of 50 and a standard deviation of 10. Here, higher values indicate a higher HRQoL. As recommended by the Word Health Organization (WHO) the well-established general health item (GHI) was additionally assessed (“In general, how would you rate your health?”) with a five-point Likert scale (from 1 = “excellent” to 5 = “poor”) (29). The KIDSCREEN-10 index yields a global HRQoL score and is recommended for use in large epidemiological surveys (25).

The KIDSCREEN-10 index previously demonstrated good internal consistency (Cronbach's alpha = 0.82) and good test-retest reliability/stability (*r* = 0.73; ICC = 0.72) (25). Additional statistical analyses showed that the KIDSCREEN-10 index is able to differentiate between groups, whereby children and adolescents with behavioral problems (SDQ, effect size Cohen's *d* = 1.30) and with a high number of psychosomatic complaints (*d* = 1.69) had significantly lower HRQoL compared to the respective control group (25).

The optimal cutoff values of Hirschfeld et al. were used to classify HRQoL, with values below this cutoff indicating poor HRQoL and values above this cutoff indicating good HRQoL. The threshold for good HRQoL was above 42.52 for children and younger adolescents (<14 years) and above 40.29 for older adolescents (≥14 years) (30).

### Epidemic-related Dark Future Scale for children (eDFS-K)

The German Likert-scaled epidemic-related Dark Future Scale for children (“**e**pidemiebezogene **D**ark **F**uture **S**cale für **K**inder”, eDFS-K) exemplified by COVID-19 was developed by Voltmer and von Salisch in 2021 and is based on the five-item Dark Future Scale by Zaleski et al., which represents a short version of the Future Anxiety Scale (16, 17, 31).

The eDFS-K measures children's future anxiety in specific relation to an epidemic (17). In the present study, direct reference was made to COVID-19, as in the validation study, but as pointed out by the authors, the scale can be adapted to any epidemic (17). The scale consists of the following four items:

(1) Are you afraid that [the Corona virus] will stay for a long time?
(2) Are you afraid that your life will get worse due to [the Corona virus]?
(3) Are you afraid that your family will soon be able to afford less due to [the Corona virus]?
(4) Are you afraid that due to [the Corona virus] you won't be able to pursue your hobbies, graduate from school or get your dream job in the future?

The 4-point Likert scale consists of the response options “Never” (0 points), “Rarely” (1 point), “Sometimes” (2 points), and “Often” (3 points). The scores of the individual items were summed up as specified by the developers, resulting in summed scores of 0–12, whereby higher scores indicate more pandemic related future anxiety. Sum scores of 0–6 were grouped as never to rarely anxious and sum scores of 7–12 were grouped as sometimes to often anxious. The developers first validated the scale on *N* = 140 third and fourth grade school children aged between 7 and 11 years in Germany (17). Since the present study applied the scale to 8–18-year-old students, the scale was validated for the respective age group.

### Psychometric analysis of the eDFS-K

The psychometric analysis was based on methods from classical test theory (CTT) and included besides item and scale characteristics the investigation of inter-item correlations and corrected item-total correlations (discriminatory power). Correlation coefficients between the items >0.30 were considered adequate, and, thus, assumed to measure the same construct (32). Reliability (internal consistency) was tested with Cronbach's α, whereby values ≥0.7 were considered acceptable (33). Furthermore, Cronbach's α was reported if one item of the eDFS-K was not included.

To verify the assumed one-factor structure (construct validity), a confirmatory factor analysis (CFA) was conducted for the total sample and additionally for subgroups divided by age (8–11 years-olds and 12–18 years-olds). Overall model fit testing was performed using χ^2^ test. The CFA was performed by robust maximum likelihood estimators (MLR) considering the root mean square error of approximation (RMSEA) with values ≤0.06, the standardized root mean square residual (SRMR) with values ≤0.08, the Tucker-Lewis index (TLI) with ≥0.95 and the comparative fit index (CFI) with ≥0.95 as acceptable model fit (32, 34). Path diagrams were used to display the standardized factor loadings and variances. Confirmatory factor analysis (CFA) was performed on R version 4.0.4 using the lavaan package version 0.6–11.

### Statistical analysis

Questionnaires were excluded from the analysis if both the eDFS-K and KIDSCREEN-10 were not completed or if students were not 8–18 years old, as the KIDSCREEN-10 is recommended and has been validated for 8–18 years-olds by the developer. The standardized questionnaires were evaluated according to the developers' specifications. The results of the KIDSCREEN-10 [the T-score calculated according to the developer's specifications (25) and the proportion of children and adolescents with low HRQoL according to cut-off values by Hirschfeld et al. (30)] were presented for the total group and subdivided by gender and in relation to the frequency of future anxiety (rarely to never vs. sometimes to often).

Nominal variables were presented with absolute and relative frequencies, whereas continuous or ordinal variables were reported with the mean and standard deviation (SD) or with the median and interquartile range (IQR) depending on presence or absence of a normal distribution. The Chi-Square (χ^2^) test was applied to compare two categorical variables. The Spearman rank correlation was performed for the comparison of at least two ordinal scaled variables. The Spearman rank correlation coefficient (Spearman Rho) is reported together with its 95% confidence interval (CI).

A general linear regression model was fitted to examine factors associated with the HRQoL (T-scores) measured by the KIDSCREEN-10 (dependent variable). The following independent variables were included in the model: Age in years, gender, school type, grade level (categorized into 1st−6th grade and 7th−13th grade), and sum of the eDFS-K. After univariable analysis all variables were considered in a multivariable model. Furthermore, interactions between variables were examined. Due to multicollinearity between the variables age in years and grade level, grade level was not considered in the two multivariable regression models. With respect to heteroscedasticity, we reported robust standard errors (HC3 estimators) (35). Marginal effect plots with the estimated values and the 95% confidence interval (CI) were generated for each of the independent variables in the multivariable regression model to illustrate the effects on HRQoL using the R package ‘ggeffects' (36, 37).

Regression coefficients (*B*) are reported with 95% CI. The goodness of fit of the model was assessed using R^2^ and the corrected R^2^. Cohen's *f*^2^ was calculated with the formula *f*^2^ = [corrected R^2^/(1-corrected R^2^)], whereby *f*^2^ ≥0.02, *f*^2^ ≥0.15, and *f*^2^ ≥0.35 represent small, medium, and large effect sizes, respectively (38). A *p*-value <0.05 was considered statistically significant. Statistical analysis was performed using IBM SPSS Statistics version 27 and R (version 4.0.4).

## Results

A total of *N* = 1,043 students participated in the web-based survey. Of these, *n* = 162 (15.5%) students did not complete the eDFS-K and KIDSCREEN-10, and another *n* = 41 (3.9%) students were outside the age range of 8–18 years.

### Sociodemographic characteristics

Overall, *N* = 840 participants were included in the analysis. The sociodemographic characteristics of the students are shown in Table 1. On average, participants were 14.8 years old, nearly 60% were female, and the majority of participants attended grammar schools (68%).

**Table 1:** Descriptive statistics for participating school children included in the study.

**Sociodemographic characteristics**	**School children**
	**(*N* = 840)**
Age in years (mean, SD)	14.8 (±2.3)
Gender, *n* (%)	
Male	320 (38.9%)
Female	491 (59.7%)
Diverse	12 (1.5%)
School type, *n* (%)	
Elementary school	41 (4.9%)
Regional school	123 (14.7%)
Grammar school	566 (67.8%)
Comprehensive school	103 (12.3%)
Special school	2 (0.2%)
Grade, *n* (%)	
1st−6th grade	107 (12.8%)
7th−13th grade	731 (87.2%)

### Psychometric analysis of the eDFS-K

The results of the psychometric analysis can be found in the supplementary data file (see Supplementary Tables 1–4 and Supplementary Figure 2). On average, the total sum score of the eDFS-K was 5.98 points, with items 1, 2 and 4 averaging between 1 and 2 points and item 3 averaging at 1 point, indicating that COVID-19 related future anxiety was rarely to sometimes present on average. Cronbach's alpha was calculated to estimate the internal consistency of the eDFS-K. The developer specified an acceptable alpha of 0.76, which we confirmed in our study (α = 0.77), indicating that the answers to the questions of the instrument are rather consistent (17). When one item was removed, there was no increase in internal consistency (α = 0.66–0.76). There was a good inter-item correlation (between *r* = 0.34 and *r* = 0.64), as well as a good corrected item-total correlation (between *r* = 0.47 and *r* = 0.66).

The construct validity of the eDFS-K was demonstrated using a confirmatory factor analysis, which indicated an acceptable model fit (χ^2^ = 56.4, df = 2, *p* < 0.001, RMSEA = 0.18, CFI = 0.94, TLI = 0.82, SRMR = 0.05), especially for 8–11 years-old students supporting that the four items of the eDFS-K measure one single construct (Supplementary Table 4). However, the RMSEA score indicated poor model fit, which might be due to the few degrees of freedom (df = 2) in this study (39). The significant *p*-value indicated a poor overall model fit, however, this is most likely due to the high sample size (40). Factor loadings for the overall model ranged from 0.49 to 0.85, with the model for 8–11 years-olds showing higher factor loadings at 0.72 to 0.81 (Supplementary Figure 2). In the model for 12–18 years-olds, item 3 indicated a lower factor loading of 0.46 compared to the other three items (0.60–0.85), which indicates that the question about fears of the future regarding the family's financial situation is less reflective of the construct of pandemic-related future anxiety among adolescents.

Content and criterion validity as well as test-retest reliability could not be determined because the eDFS-K was due to the cross-sectional study design queried only once, no additional instrument was used, and no experts were consulted.

### COVID-19-related future anxiety (eDFS-K)

The results of the four-item eDFS-K are shown in Table 2. The distribution of responses for items 1, 2, and 4 were comparable. 20–21% of respondents never feared that the Corona virus will stay for a long time, that life will deteriorate as a result of the Corona virus, or that they will not be able to pursue their hobbies, graduate from school, or gain their dream job, whereas 24–28% often feared such things. The fear that the family will be able to afford less due to Corona virus was often feared by 13% of participants, while 39% never had this fear.

**Table 2:** Results of the epidemic-related Dark Future Scale for children (eDFS-K).

**Item no**.	**Epidemic-related Dark Future Scale for children (*N* = 826)**	**Never**	**Rarely**	**Sometimes**	**Often**
1	Are you afraid that the Corona virus will stay for a long time?	174 (21.1%)	163 (19.7%)	288 (34.9%)	201 (24.3%)
2	Are you afraid that your life will get worse due to the Corona virus?	171 (20.7%)	176 (21.3%)	277 (33.5%)	202 (24.5%)
3	Are you afraid that your family will soon be able to afford less due to the Corona virus?	325 (39.3%)	218 (26.4%)	177 (21.4%)	106 (12.8%)
4	Are you afraid that due to the Corona virus you won't be able to pursue your hobbies, graduate from school or get your dream job in the future?	163 (19.7%)	182 (22.0%)	250 (30.3%)	231 (28.0%)

Overall, 46.5% of the students reported sometimes to often COVID-19-related fears about the future, with a significantly higher proportion of females reporting frequent fears about the future compared to males and participants with diverse gender (females: *n* = 272, 56.7%; males: *n* = 97, 30.6%; diverse: *n* = 4, 33.3%; *p* < 0.001). There were no age differences between respondents who indicated more or less frequent fears about the future (never to rarely: 14.8 ± 2.2 years; sometimes to often: 14.9 ± 2.5 years; *p* = 0.169). However, elementary school students were found to have significantly higher eDFS-K sum scores (*n* = 41, median = 8.0, IQR = 6.5) than students from grammar schools (*n* = 554, median = 6.0, IQR: 4.0; *p* = 0.010) regional schools (*n* = 121, median = 6.0, IQR = 6.0; *p* = 0.035), and comprehensive schools (*n* = 103, median = 6.0, IQR = 5.0; *p* = 0.003). There were no significant differences in eDFS-K sum scores between students from grammar schools, regional schools, and comprehensive schools (grammar vs. regional school, *p* = 0.928; grammar vs. comprehensive school, *p* = 0.231, regional vs. comprehensive school, *p* = 0.307).

### Health-related quality of life (KIDSCREEN-10)

Overall, 43.5% of students had low HRQoL, whereby the proportion of girls with low HRQoL was significantly higher than the proportion of boys (52.6 vs. 28.9%, *p* < 0.001, Table 3). When considering HRQoL subdivided by age group, 34.3% of children under 12 years of age and 44.7% of children 12 years of age and older showed low HRQoL (*p* = 0.094). Furthermore, it was found that the HRQoL was significantly lower in children with more frequent future anxiety (sometimes to often) than in children with infrequent future anxiety (never to rarely) (59.1% vs. 30.8%, *p* < 0.001). Boys with no to rare future anxiety reported the highest HRQoL (normal/high HRQoL = 78.7%) and girls with more frequent future anxiety had the lowest HRQoL (normal/high HRQoL = 36.6%).

**Table 3:** Results of the self-reported KIDSCREEN-10.

	**All 8–18 years old's**			**Never to rarely afraid children (eDFS-K)**			**Sometimes to often afraid children (eDFS-K)**			***p-*value^#^**
	***N***	**TS**	Low HRQoL*(%)	***N***	**TS**	Low HRQoL*(%)	***N***	**TS**	Low HRQoL*(%)	
Male	311	46.2	28.9	216	48.0	21.3	93	42.3	45.2	<0.001
Female	481	41.5	52.6	205	43.7	40.5	265	39.4	63.4	<0.001
Diverse	12	38.3	58.3	8	40.6	37.5	4	33.8	100	–
Total	804	43.3	43.5	429	45.8	30.8	362	40.1	59.1	<0.001

TS, mean values of T-score of KIDSCREEN-10.

^*^Low HRQoL according to cutoff values by Hirschfeld et al. (30).

^#^*p*-values resulting from χ^2^ test comparing low vs. normal/high HRQoL between eDFS-K groups (never to rarely afraid children vs. sometimes to often afraid children).

### Association of HRQoL and COVID-19-related future anxiety

All items of the KIDSCREEN-10 were negatively correlated with the eDFS-K (from *r* = – 0.18 to *r* = – 0.29; *p* < 0.001) except for the two items that have to be reversed for the calculation of the overall HRQoL score (item 3, *r* = 0.35; item 4, *r* = 0.36; *p* < 0.001), as for these items higher scores indicate lower HRQoL (see Supplementary Table 5). Furthermore, the negative association between the sum score of the eDFS-K and the HRQoL overall score was robust across age groups subdivided by 2-year intervals (3-year interval for 8–10 years-olds due to small sample sizes), respectively (*r* = – 0.36 to *r* = – 0.49, *p* ≤ 0.004; see Supplementary Table 6).

To further investigate factors that influence HRQoL, a general linear regression model was fitted (Table 4). Participants attending special schools were not included in the regression models due to the small sample size. In the univariable models, older age (*B* = – 0.8, *p* < 0.001), female (*B* = – 4.8, *p* < 0.001) and diverse (*B* = – 7.9, *p* < 0.001) gender, 7th−13th grade attendance (*B* = −3.9, *p* < 0.001), and more frequent future anxiety (*B* = – 1.1, *p* < 0.001) were associated with decreasing HRQoL. Elementary (*B* = 4.6, *p* = 0.010), regional (*B* = 2.8, *p* = 0.003), and comprehensive school students (*B* = 2.8, *p* = 0.001) had higher HRQoL compared to grammar school students.

**Table 4:** General linear regression models with health-related quality of life (self-reported KIDSCREEN-10) as dependent variable, *N* = 787.

**Variable**	**Univariable model**		**Multivariable model 1**		**Multivariable model 2**	
	***B* (95% CI)**	***p*-value**	***B* (95% CI)**	***p*-value**	***B* (95% CI)**	***p*-value**
Intercept			59.62 (54.89, 64.34)	**<0.001**	55.03 (47.80, 62.27)	**<0.001**
Age						
in years	– 0.766 (– 1.034, – 0.498)	**<0.001**	– 0.627 (– 0.917,−0.336)	**<0.001**	– 0.324 (– 0.787, 0.140)	0.171
Gender						
Male	ref.		ref.		ref.	
Female	– 4.809 (– 6.031, −3.587)	**<0.001**	– 3.116 (– 4.281,−1.950)	**<0.001**	4.248 (– 3.729, 12.226)	0.296
Diverse	– 7.888 (– 11.490, – 4.285)	**<0.001**	– 6.819 (– 10.283, – 3.356)	**<0.001**	– 13.224 (– 35.502, 9.053)	0.244
Age*Gender						
Age*Female	– 0.597 (– 1.152, – 0.042)	**0.035**	**–**	–	– 0.497 (−1.015, 0.021)	0.060
Age*Diverse	0.598 (– 2.265, 3.460)	0.682	–	**–**	0.430 (– 1.048, 1.908)	0.568
School type						
Grammar school	ref.		ref.		ref.	
Elementary school	4.599 (1.113, 8.085)	**0.010**	1.366 (– 2.551, 5.284)	0.494	1.890 (– 2.077, 5.857)	0.350
Regional school	2.802 (0.982, 4.622)	**0.003**	1.561 (– 0.217, 3.340)	0.085	1.589 (– 0.187, 3.365)	0.079
Comprehensive school	2.792 (1.076, 4.509)	**0.001**	1.122 (– 0.493, 2.738)	0.173	1.146 (– 0.470, 2.762)	0.164
Grade						
1st−6th grade	ref.					
7th−13th grade	– 3.871 (– 5.794, – 1.948)	**<0.001**	–	–	–	–
Dark future scale						
Sum (0–12 points)	– 1.124 (– 1.314, – 0.934)	**<0.001**	– 0.944 (– 1.144, – 0.743)	**<0.001**	– 0.934 (– 1.133, – 0.735)	**<0.001**

Ref., reference group. *p*-values <0.05 are printed in bold.

Thereafter we examined interactions between variables. The negative association between HRQoL and age in years was stronger in females than in males (*B* = – 0.6, *p* = 0.035). We did not find any interactions between eDFS-K sum and sociodemographic variables, such as age, gender, and school type.

**Figure 1:**
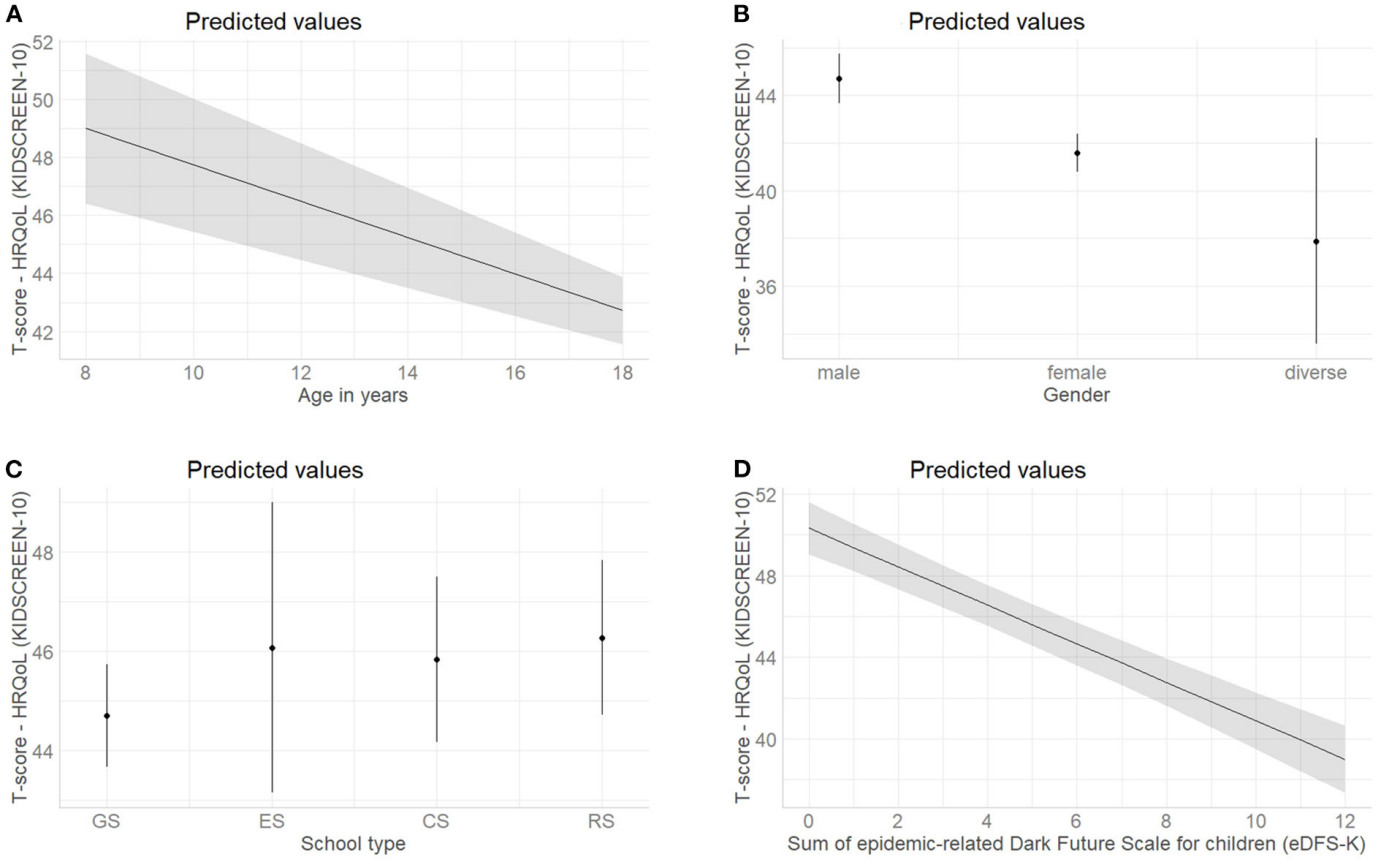
Marginal effect plots with the predicted values and the 95% confidence interval of health-related quality of life (self-reported KIDSCREEN-10) for all predictors included in the multivariable model 1 (see Table 4, *N* = 787); **(A)** Estimated effect of age in years; **(B)** Estimated effect of gender; **(C)** Estimated effect of the school type, Abbr.: GS, grammar school; ES, elementary school; CS, comprehensive school; RS, regional school; **(D)** Estimated effect of sum of epidemic-related Dark Future Scale for children (eDFS-K). For continuous predictors, the gray lines and gray areas represent the estimated effect and 95% confidence interval, while for nominal variables this is represented by dots and dashes.

The multivariable model 1 without considering any interactions showed a decrease in HRQoL with increasing age (*B* = – 0.6, *p* < 0.001), female (*B* = – 3.1, *p* < 0.001) and diverse (*B* = – 6.8, *p* < 0.001) gender, and more frequent COVID-19-related future anxiety (*B* = – 0.9, *p* < 0.001). The school type was no longer associated with HRQoL. Grade level was not included in the multivariable model due to multicollinearity with age. Overall, the model accounted for 24% of the variance with a medium effect size (R^2^ = 0.248, corrected R^2^ = 0.242, Cohens *f*^2^ = 0.32). Estimated values with the respective 95% confidence interval of HRQoL are illustrated in Figures 1A–D for all predictors in the multivariable model 1.

**Figure 2:**
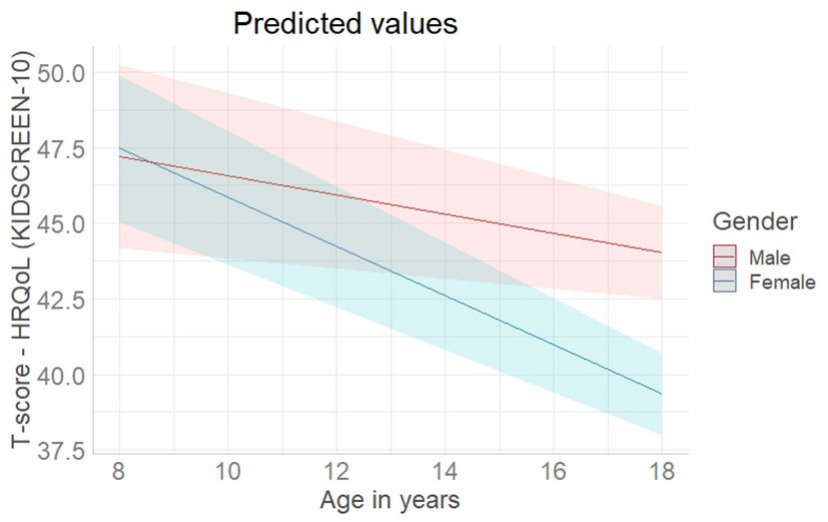
Interaction plot of age in years and female gender in relation to health-related quality of life (self-reported KIDSCREEN-10. Adjusted for school type and sum of epidemic-related Dark Future Scale for children (eDFS-K) (see Table 4, Multivariable Model 2). The colored lines and the colored areas represent the estimated effects and the 95% confidence intervals.

After adding the interaction term between age and gender to the model (Table 4, Multivariable Model 2), age as well as female and diverse gender alone were no longer associated with HRQoL, and the interaction between age and female gender trended toward significance (*B* = – 0.5, *p* = 0.060). An interaction plot illustrates the interaction of gender and age in years on HRQoL (Figure 2). The regression coefficient of the sum score of the eDFS-K was comparable to the multivariable model 1. Model 2 also accounted for 24% of the variance with a medium effect size (Model 2: R^2^ = 0.253, corrected R^2^ = 0.244, Cohens *f*^2^ = 0.32). Overall, the interaction term did not contribute significantly to the predictive power of the model.

## Discussion

In the context of the COVID-19 pandemic, previous studies have found significant burdens on children and adolescents, with adverse effects on the mental and physical health (1–3, 15). To the best of our knowledge the present study is the first to examine COVID-19-related future anxiety two years after the onset of the pandemic in over 800 8–18 years-old children and adolescents using the eDFS-K in terms of its association with HRQoL. It was shown that more than 40% of the children and adolescents still have a low HRQoL 2 years after the onset of the COVID-19 pandemic and more than 45% sometimes to often fear that life will deteriorate due to the Corona virus and that hobbies, school graduation or the dream job can no longer be pursued or achieved. More frequent COVID-19-related future anxiety was associated with lower HRQoL.

As part of the study, a psychometric analysis of the eDFS-K was conducted, based on the application of the instrument among children and adolescents aged 8–18 years. So far, the instrument has only been evaluated for children aged 7–11 years (17). On the one hand, we were able to confirm the developer's results for 8- to 11-year-olds (17), and on the other hand to demonstrate comparable validity and reliability for 12–18 years-olds. Only item 3 of the eDFS-K, asking about future anxiety in relation to the family's financial situation, reflected the one-factor construct of pandemic-related future anxiety to a lesser extent for 12–18 years old school students than for 8–11 years old school children. One reason for this might be a more differentiated view of the financial situation with increasing age. Two years after the onset of the pandemic, fewer occupational and thus financial changes in the family were to be expected, and our results indicate that this situation could be better assessed by older children. Despite a somewhat poorer model fit for the 12–18 years-olds compared to the 8–11 years old school students, our results largely confirm the findings with respect to internal consistency reliability and construct validity of the eDFS-K from the previous validation study (17), indicating that the instrument can be used to assess COVID-19-related fears about the future among children and adolescents.

In Germany, a recent representative nationwide study in 7–17 years-old children and adolescents has been conducted by Ravens-Sieberer et al. as part of the COPSY study (COVID-19 and Psychological Health). In their first analysis, cross-sectional data collected between May and June 2020 were compared to pre-pandemic data from the nationally representative BELLA study (Behavior and Well-being of Children and Adolescents in Germany) (13). In both studies HRQoL was also assessed using the KIDSCREEN-10. In the COPSY study the proportion of children with low HRQoL was 40.2% overall, 44.7% in girls, and 35.7% in boys (13). A significant increase in the proportion of children with a low HRQoL compared to before the pandemic was observed. Subsequently, the Corona cohort was surveyed again between December 2020 and January 2021 (12). At this time the proportion of children and adolescents with a low HRQoL had further increased to 47.7%. This difference compared to the first Corona cohort, however, was not statistically significant (12).

Overall, the proportion of children and adolescents with a low HRQoL after 2 years of the pandemic remains high at almost 44% compared to data from the COPSY study from South Tyrol (Italy) and Germany from May–June 2021 and September–October 2021, respectively, where the overall self-reported low HRQoL rate of children and adolescents was 33–35% (15, 41). One possible cause for the increase of the population with a low HRQoL in our study could be the intensification of restriction measures in schools in the study region over the winter of 2021 and spring of 2022 due to high SARS-CoV-2 incidence rates, which was accompanied by mandatory masking, and mandatory testing in schools (3x per week). In Swiss primary school children, it was also found that HRQoL deteriorated at the heights of the COVID-19 waves, however, it could not be determined whether fear of the disease or the restrictions caused this decrease in HRQoL (42).

Furthermore, our study showed a decrease in HRQoL with increasing age and for female and diverse gender. We found that in terms of HRQoL, age and gender interact: For females, HRQoL tended to decrease more strongly with age compared to males. Barbieri et al. observed that the proportion of children in South Tyrol with low HRQoL was higher in girls compared to boys (38 vs. 28%) (41). Another recent study from Germany also reported such age and gender differences (43). However, these findings are not surprising. In previous studies conducted before the onset of the COVID-19 pandemic, gender and age differences in HRQoL were found, whereby a higher HRQoL was found in younger than in older and in male compared to female participants; the age-specific difference was more pronounced in girls (44–46). The reasons for these gender- and age-related differences have not yet been conclusively clarified (47). Increasing pressure on girls and boys with the onset of puberty is thought to play a crucial role in the age-related differences (48). Furthermore, it has been found that girls face more stressful events during the transition to adolescence than boys and show a stronger maladaptive coping pattern—in particular with regard to social stressors (49). Hormonal changes are also discussed as causative factor for the gender-related differences (49, 50). Gender- and age-related differences are also evident in the prevalence of a range of mental health problems and subjective wellbeing, with girls being more frequently affected than boys (49, 51).

In addition to HRQoL, mental health problems, as well as anxiety and depression, were also examined during the COVID-19 pandemic by Ravens-Sieberer et al. (12). The proportion of children and adolescents with mental health problems (17.6% pre-Corona vs. 30.4% in the first Corona cohort and 30.9% in the second Corona cohort), anxiety symptoms (14.9% pre-Corona vs. 24.1% and 30.1%), and depressive symptoms (10.0% pre-Corona vs. 11.3% and 15.1%) also increased after the onset of the pandemic (12). Girls reported depressive symptoms (females 20.2%, males 10.3%) and generalized anxiety symptoms (females 34.6%, males 19.2%) more frequently than boys (41). In the validation study of the eDFS-K, girls showed more pronounced COVID-19-related future anxiety than boys, which is also consistent with the findings of the present study (17). With respect to age-related differences, a different trend was observed in the present study than for HRQoL: COVID-19-related fears about the future peaked among 8–10-year old's, then decreased until 13–14 years of age, and then increased again until late adolescence. These results however should be interpreted with caution, as future anxiety was not longitudinally assessed, the participation rate of 8- to 10-year-olds was low and no reference values are available.

Interestingly, a study by Van Oort et al. who longitudinally assessed general anxiety symptoms of 2200 boys and girls showed a similar pattern: They found that anxiety symptoms first decrease during early adolescence, and subsequently increase from middle to late adolescence (52). Similar findings were also reported in another longitudinal study by Cohen et al. with anxiety symptoms decreasing until age 12 (the “developmental knot”) and then increasing into early adolescence (53). They hypothesized that the often stressful transition from childhood to early adolescence, along with changing life circumstances, such as most children transfer from elementary to secondary school, may be reflected in initially higher anxiety scores in late childhood (52, 53). In later adolescence, as the children mature into autonomous, independent individuals, adult expectations increase, and feelings of insecurity and worry during this time may explain the increase in anxiety (52, 53). With regard to COVID-19-related future anxiety, one might hypothesize that such processes may also contribute to increased anxiety about the future. However, in the validation study by Voltmer et al. a trend toward an increase in COVID-19 related future anxiety was reported in 7–11 years-old elementary school students [*r* = 0.15, *p* = 0.074, (17)]. Ultimately, further longitudinal studies with sufficient power are needed to determine age-related differences with regard to future anxiety in children and adolescents.

Our results indicate that two years after the onset of the pandemic the mental burdens of the COVID-19 pandemic remain persistently high and that the pandemic management in Germany does not seem to be effective in addressing them. A need for psychosocial support for children in Germany was registered by scientists and the government and recommendations for action were postulated; concepts for mental health care promotion in children and adolescents with specific aims, however, have not yet been defined (54, 55).

Our results add to the growing body of evidence showing that psychosocial support during an outbreak is not less important than infection control (56). In particular, multidisciplinary support by professionals such as psychiatrists, psychologists, social workers, and pediatricians is needed during a pandemic, which requires a structured and organized program, especially with regard to future pandemics, as described, e.g., by Hyun et al. (56). Thus, psychological counseling and guidance services should be expanded to help children and adolescents to better cope and regain a healthy psychological structure. Parents should also pay more attention to children's mental health, working together with teachers and experts to identify and specifically address mental problems and future anxiety. Therefore, short-term objectives for mental health care promotion should be to provide specific information about research findings on the impact of the COVID-19 pandemic on child and adolescent psychosocial health and guidance for parents and stakeholders of schools (teachers and school principals), e.g., the importance of talking with a trusted person about fears and anxieties related to the COVID-19 pandemic and associated negative feelings. This should be supported by policy-makers to ensure that it has a widespread outreach to parents and schools. Health care providers also play an essential role in educating families about how to talk to children about COVID-19 at home (57).

Furthermore, the results obtained indicate the importance of implementing intervention measures, e.g., low-threshold measures in family and school settings such as relaxation programs to reduce stress and prevention programs to strengthen resilience. Besides the increased incidence of symptoms of depressiveness and anxiety, fears about the future are also an important target for intervention programs. Therefore, long-term objectives for mental health care promotion should contain the implementation of school-based mental health promotion programs into standard educational practice to enhance resilience and coping skills that have been shown to positively impact the student's ability to manage daily stressors (58–60). However, implementing such programs into everyday life requires considerable time and could, e.g., first be examined for feasibility and acceptance as part of pilot projects in model regions. Since the burden on schools has increased during the pandemic, e.g., due to illness-related staff absences and canceled classes, time and personnel resources must also be available for such important intervention programs, which should be supported by the respective state governments. In addition, previous studies have indicated that psychosocial distress is negatively associated with academic achievement (61, 62). Our study also showed a negative association between perceived academic achievement and the frequency of anxiety about the future, further highlighting the need for action.

It is noteworthy that we were able to show for the first time that frequent COVID-19-related future anxiety is associated with lower HRQoL. However, a differentiation between future anxiety as assessed and anxiety disorders was not possible in the context of this study and needs to be further evaluated in further studies. It would also be of interest to assess stress perception, self-efficacy, and coping skills, and to examine the impact on future anxiety.

A limitation of this survey is the cross-sectional design, whereby the results only represent one point in time. Further, no pre-pandemic results were available for our setting, which would have allowed a direct comparison. Another important limitation is the use of an instrument that has so far been rarely used in studies to measure COVID-19-related future anxiety in children and adolescents. We were able to show that construct validity and internal consistency reliability were also high in children 12 years and older. However, content and criterion validity as well as test-retest reliability could not be determined, thus further studies are needed for a comprehensive psychometric analysis of the eDFS-K in children and adolescents. Also, the survey may not be representative because the response rate of the students was comparatively low (<1% of all students in the study region participated), adolescents from grammar schools were overrepresented and the study region was limited to one Federal State in Germany. We do not see, however, clear indication for any structural difference between Mecklenburg-Western Pomerania and other Federal States with respect of the perception of the COVID-19 pandemic and its possible impact on the future among school children. Moreover, comparable results regarding the HRQoL have also been published in other countries (21, 63). In addition, it should be mentioned that 15% of the participants did not complete the questionnaire. Based on the available data, we cannot make any assumptions about the reasons for dropping out of the survey. One could assume that the content of the questionnaire, which was primarily related to psychosocial health, was perceived as too personal by some participants, or some people only wanted to take a look out of interest and did not intend to participate in the survey. Another limitation was that self-reported questionnaires were used, so the participation of children under 12 years of age was low. In addition, it cannot be conclusively determined whether the students completed the questionnaire independently or with the help of another person. The survey was conducted exclusively online and not paper-based, so that children and families without technical equipment might not have been able to participate.

In conclusion, our results further support the findings from the previous validation study suggesting that the eDFS-K can be used as an assessment tool measuring COVID-19-related future anxiety in children and adolescents aged 8 to 18 years. Future anxiety in children and adolescents with regard to HRQoL has to our knowledge not been studied so far. It was shown that frequent COVID-19-related future anxiety was associated with a lower HRQoL. Addressing future anxiety in children and adolescents should become a prime target in future intervention programs to alleviate the impact of the pandemic on the young generation. Further studies are needed to investigate future anxiety in children and adolescents in a more differentiated manner.

## Data availability statement

The raw data supporting the conclusions of this article will be made available by the authors, without undue reservation.

## Ethics statement

The study was reviewed and approved by the Ethics Committee of the University Medicine Greifswald (BB 163/21). By participating in the survey, respondents confirmed that the legal guardian/next of kin consented to participate in this study.

## Author contributions

Conceptualization and methodology: AK, PL, and WH. Data analysis: AK, PL, AH, LS, and JL. Writing—original draft preparation: AK. Writing—review and editing: PL, AH, LS, JL, and WH. Funding acquisition: WH. All authors have read and agreed to the published version of the manuscript.

## Funding

This study was financially supported by the former Ministry of Economic Affairs, Labor and Health Mecklenburg-Western Pomerania (Grant number: 400-00000-2014/107-016). The funder had no role in study design, data collection and analysis, decision to publish, or preparation of the manuscript.

## Conflict of interest

The authors declare that the research was conducted in the absence of any commercial or financial relationships that could be construed as a potential conflict of interest.

## Publisher's note

All claims expressed in this article are solely those of the authors and do not necessarily represent those of their affiliated organizations, or those of the publisher, the editors and the reviewers. Any product that may be evaluated in this article, or claim that may be made by its manufacturer, is not guaranteed or endorsed by the publisher.
