# Supplementary material for: COVID-19-related future anxiety is associated with the health-related quality of life in school-aged children and adolescents—A cross-sectional study
Source: Front Public Health. 2022 Nov 9;10:1003876. doi: 10.3389/fpubh.2022.1003876 (PMC9682149; doi:10.3389/fpubh.2022.1003876)
Supplement: Supplementary file 1 [file Data_Sheet_1.DOCX]

***Supplementary Material***

**Supplementary Table 1 –** Scale characteristics of the epidemic-related Dark Future Scale for children (eDFS-K) according to classical test theory (CTT)

| ***N*=826** | **Items** | **M** | **SD** | **skewness** | **kurtosis** | **min.- max.** | ***α*** |
| --- | --- | --- | --- | --- | --- | --- | --- |
| eDFS-K sum score | 4 | 5.98 | 3.39 | -0.09 | -0.89 | 0-12 | 0.77 |

M=mean, SD=standard deviation, min=minimum; max=maximum; α=Cohens α

**Supplementary Table 2 -** Item characteristics of the epidemic-related Dark Future Scale for children (eDFS-K) according to classical test theory (CTT)

| ***N*=826** | **M** | **SD** | **skewness** | **kurtosis** | **discriminatory power** | **Cronbach’s α when Item not included** |
| --- | --- | --- | --- | --- | --- | --- |
| Item 1 | 1.62 | 1.07 | -0.25 | -1.18 | 0.57 | 0.71 |
| Item 2 | 1.62 | 1.07 | -0.22 | -1.19 | 0.66 | 0.66 |
| Item 3 | 1.08 | 1.06 | 0.50 | -1.04 | 0.47 | 0.76 |
| Item 4 | 1.66 | 1.09 | -0.24 | -1.23 | 0.57 | 0.71 |

M=mean, SD=standard deviation

**Supplementary Table 3 –** Inter-item and item-total correlation of the epidemic-related Dark Future Scale for children (eDFS-K)

| **Item** | **1** | **2** | **3** | **4** |
| --- | --- | --- | --- | --- |
| 1 | 1 |  |  |  |
| 2 | 0.637** | 1 |  |  |
| 3 | 0.341** | 0.380** | 1 |  |
| 4 | 0.393** | 0.512** | 0.453** | 1 |
| **Item 1-4** | 0.767** | 0.822** | 0.704** | 0.768** |

Note: *0.01≤p<0.05, ***p*<0.01

**Supplementary Table 4 –** Confirmatory factor analysis (CFA) of the epidemic-related Dark Future Scale for children (eDFS-K) for all children and differentiated by 8- to 11-year-olds and 12- to 18-year-olds

|  | **x²/df/*p*** | **RMSEA** | **CFI** | **TLI** | **SRMR** |
| --- | --- | --- | --- | --- | --- |
| **All (N=826)** | 56.36/2/<0.001 | 0.18 | 0.94 | 0.82 | 0.05 |
| ***Age*** |  |  |  |  |  |
| 8- to 11-year-olds (N=70) | 2.26/2/0.324 | 0.05 | 1.00 | 0.99 | 0.03 |
| 12- to 18-year-olds (N=743) | 54.0/2/<0.001 | 0.19 | 0.94 | 0.81 | 0.06 |

RMSEA=root mean square error of approximation, SRMR=the standardized root mean square residual, TLI=Tucker-Lewis index, CFI=comparative fit index

**Supplementary Table 5 –** Results of Spearman's rank correlation for the association between the sum of the epidemic-related Dark Future Scale for children (eDFS-K) and the individual KIDSCREEN-10 items

| **KIDSCREEN-10 items (*N*=820)** | | | **Spearman’s Rho (*r*_s_)** | **95%CI** | | ***p*-value** | |
| --- | --- | --- | --- | --- | --- | --- | --- |
| 1 | Have you felt fit and well? | -0.268 | | -0.332, -0.201 | <0.001 | |  |
| 2 | Have you felt full of energy? | -0.237 | | -0.302, -0.169 | <0.001 | |  |
| 3 | Have you felt sad? | 0.353 | | 0.290, 0.413 | <0.001 | |  |
| 4 | Have you felt lonely? | 0.364 | | 0.302, 0.424 | <0.001 | |  |
| 5 | Have you had enough time for yourself? | -0.257 | | -0.322, -0.190 | <0.001 | |  |
| 6 | Have you been able to do the things that you want to do in your free time? | -0.291 | | -0.354, -0.225 | <0.001 | |  |
| 7 | Have your parent(s) treated you fairly? | -0.184 | | -0.252, -0.115 | <0.001 | |  |
| 8 | Have you had fun with your friends? | -0.217 | | -0.284, -0.149 | <0.001 | |  |
| 9 | Have you got on well at school? | -0.256 | | -0.321, -0.189 | <0.001 | |  |
| 10 | Have you been able to pay attention? | -0.293 | | -0.356, -0.227 | <0.001 | |  |

CI=confidence interval

**Supplementary Table 6** - Results of the Spearman's rank correlation for the association between the sum of the epidemic-related Dark Future Scale for children (eDFS-K) and HRQoL (KIDSCREEN-10) divided by age

|  | ***N*** | **eDFS-K (median, IQR)** | **HRQoL (mean, SD)** | **Spearman’s Rho (*r*_s_)** | **95%CI** | ***p*-value** |
| --- | --- | --- | --- | --- | --- | --- |
| ***Age*** |  |  |  |  |  |  |
| 8- to 10-years | 49 | 8 (6.5) | 46.8 (±10.3) | -0.400 | -0.617, -0.125 | 0.004 |
| 11- to 12-years | 71 | 6 (6) | 45.8 (±8.8) | -0.490 | -0.653, -0.284 | <0.001 |
| 13- to 14-years | 211 | 5 (6) | 44.9 (±8.8) | -0.387 | -0.500, -0.263 | <0.001 |
| 15- to 16-years | 252 | 6 (4) | 41.9 (±9.0) | -0.400 | -0.502, -0.287 | <0.001 |
| 17- to 18-years | 224 | 7 (5) | 41.3 (±7.5) | -0.356 | -0.469, -0.233 | <0.001 |

IQR=interquartile range, SD=standard deviation, CI=confidence interval

*
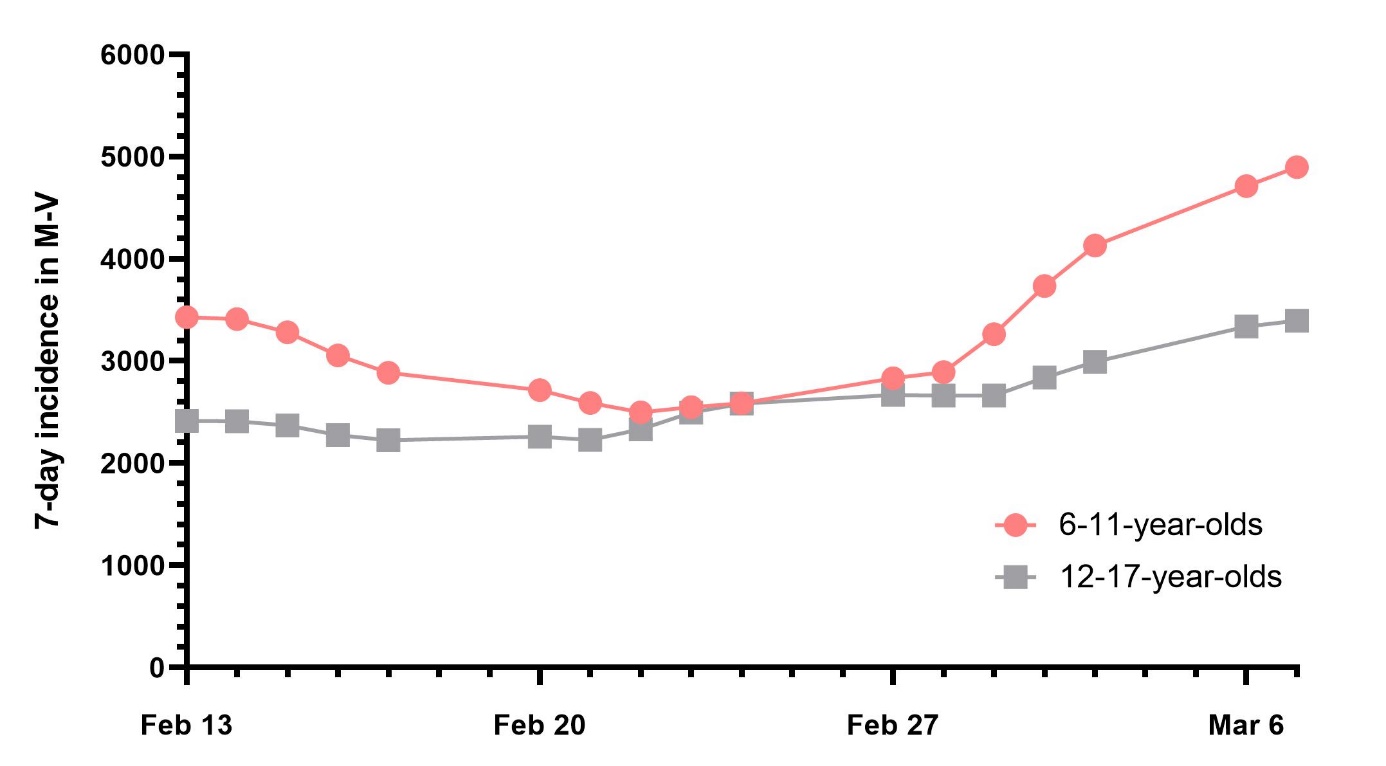
*

**Supplementary Figure 1 –**7-day-incidence per 100,000 residents of PCR-confirmed SARS-CoV-2 infections in the study region (Mecklenburg-Western Pomerania) during the study period in 2022 divided into the age groups of 6-11-year old’s and 12-17-year old’s; Reference: Daten Zur Corona-Pandemie - Archiv Für Die Altersspezifischen Lageberichte Kinder Und Jugendliche [Internet]. (2022) [cited 2022-06-23]. Available from: https://www.lagus.mv-regierung.de/Gesundheit/InfektionsschutzPraevention/Daten-Corona-Pandemie.


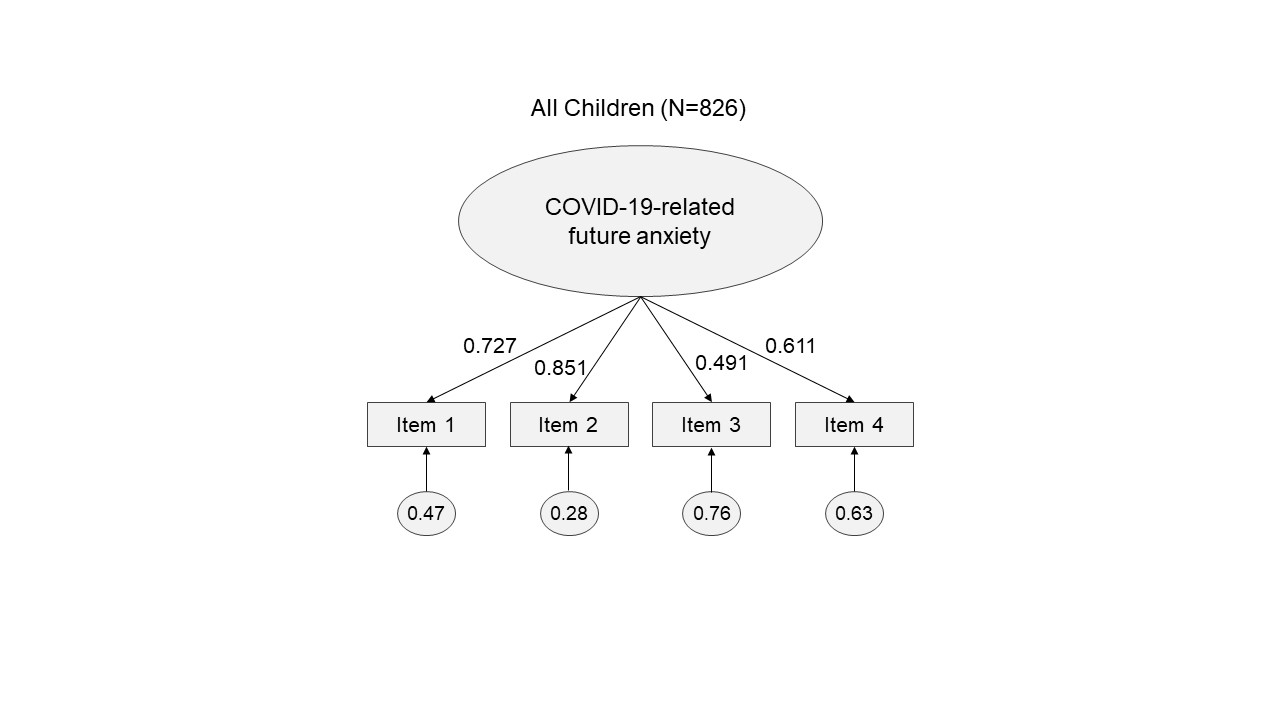

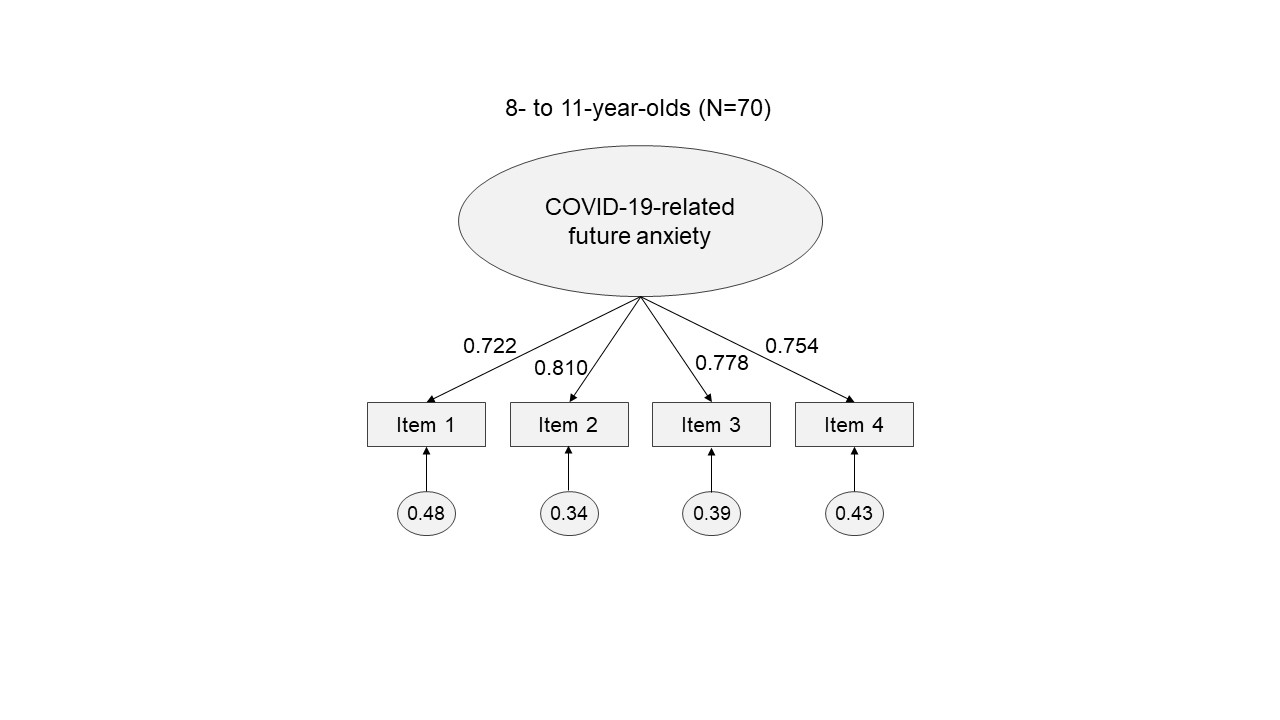

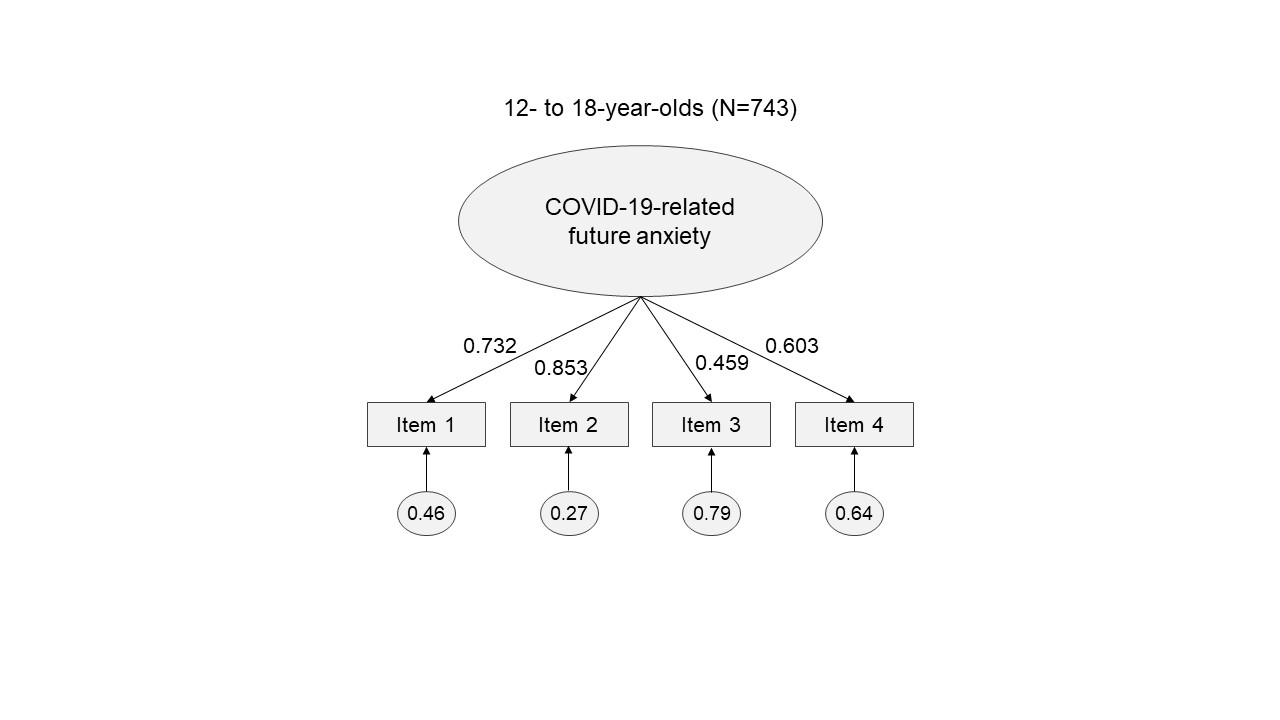


**Supplementary Figure 2** – Path diagrams of the confirmatory factor analysis (CFA) of the epidemic-related Dark Future Scale for children (eDFS-K) for all children (left) and differentiated by 8- to 11-year-olds (center) and 12- to 18-year-olds (right)
